# Supplementary material for: Novel Hits for N-Myristoyltransferase Inhibition Discovered by Docking-Based Screening
Source: Molecules. 2022 Aug 26;27(17):5478. doi: 10.3390/molecules27175478 (PMC9457982; doi:10.3390/molecules27175478)
Supplement: Supplementary file 1 [file molecules-27-05478-s001.zip › molecules-1867461-supplementary.pdf]

Supplementary Materials

Novel Hits for N-Myristoyltransferase Inhibition Discovered by Docking-Based Screening

Danislav S. Spassov *, Mariyana Atanasova and Irini Doytchinova

Department of Chemistry, Faculty of Pharmacy, Medical University of Sofia, 1000 Sofia, Bulgaria
* Correspondence: dspassov@pharmfac.mu-sofia.bg

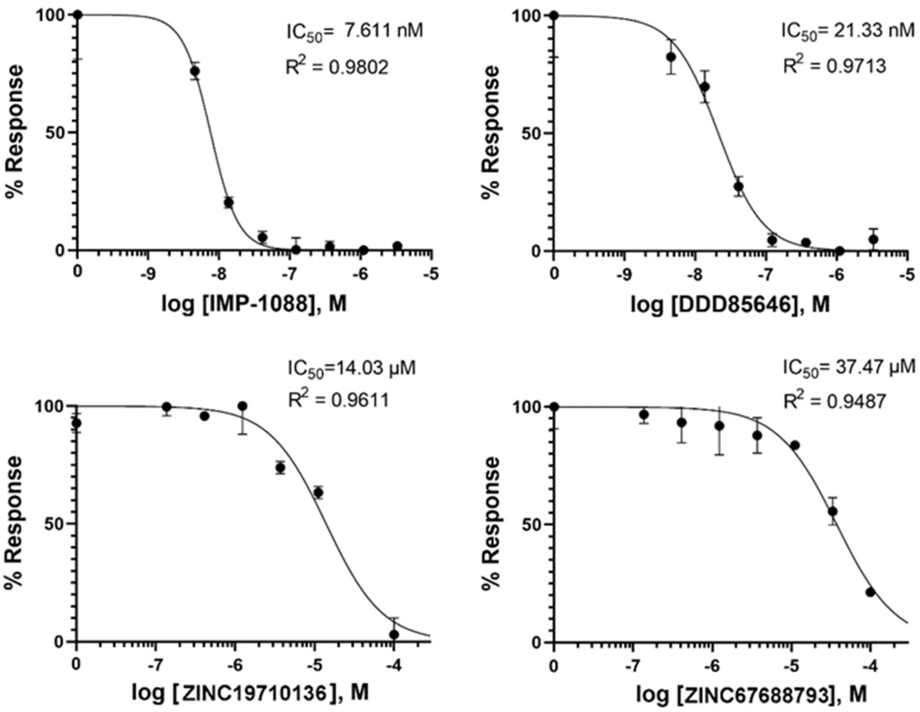

Figure S1. Dose response inhibitory curves and IC_{50} of selected compounds. The control NMT inhibitors are shown on the top row. At the bottom, two ligands- ZINC19710136 and ZINC67688793, identified by virtual screening are shown. These compounds form a salt bridge with C-terminus of NMT and were some of the most potent ligands tested. The experiments were performed in duplicates. Error bars represent SEM.

Table S1. ChemPLP scores of candidate compounds after docking with GOLD using crystal structure of human NMT1 (PDB 3IWE). The protein was set with flexible side chains. Three structural water molecules (HOH2, HOH760, HOH970) were also included in analysis. Autodock affinity scores are also shown for comparison.

	ZINC ID	ChemPLP GOLD	Affinity Autodock kcal/mol	Interaction with C-terminus of NMT
1	ZINC19710084	120.37	-11.4	Salt bridge
2	ZINC19691948	120.17	-12.0	H-bond
3	ZINC19710136	119.73	-11.6	Salt bridge
4	ZINC19710116	117.55	-11.3	Salt bridge
5	ZINC19228549	117.52	-11.9	None
6	ZINC15080181	116.02	-11.1	Salt bridge
7	ZINC19710117	115.26	-11.3	Salt bridge
8	ZINC35451413	114.82	-11.8	H-bond
9	ZINC21711800	113.94	-11.3	Salt bridge
10	ZINC61997750	113.09	-11.9	None

11	ZINC46055559	112.95	-11.9	H-bond
12	ZINC35458799	112.31	-11.3	Salt bridge
13	ZINC19692196	111.64	-11.8	H-bond
14	ZINC20600095	111.56	-11.9	None
ctr	IMP-1088	111.36	-10.8	Salt bridge
15	ZINC19692195	110.28	-12.0	H-bond
16	ZINC65111278	110.24	-11.6	None
17	ZINC19590209	110.20	-11.9	Salt bridge
ctr	DDD86481	110.20	-11.6	Salt bridge
18	ZINC214463354	110.07	-12.0	Salt bridge
19	ZINC19781807	109.50	-11.7	None
20	ZINC34830480	109.37	-11.7	None
21	ZINC95642832	108.84	-12.0	None
22	ZINC306129675	108.58	-11.8	None
23	ZINC257281908	108.43	-11.6	H-bond
24	ZINC19347015	108.35	-11.6	H-bond
25	ZINC118915711	108.08	-11.7	Salt bridge
26	ZINC8992179	108.00	-11.6	None
27	ZINC257217479	107.87	-11.9	None
28	ZINC257333200	107.67	-11.8	None
29	ZINC19376075	107.51	-12.2	H-bond
30	ZINC257338200	107.46	-11.7	None
31	ZINC15240419	107.44	-11.7	None
32	ZINC61997749	107.33	-11.8	None
33	ZINC257248718	107.29	-11.9	None
34	ZINC61999344	107.07	-11.8	None
35	ZINC299757951	106.95	-11.5	Salt bridge
36	ZINC3854978	106.67	-11.7	None
37	ZINC19692188	106.62	-11.6	H-bond
38	ZINC12047789	106.57	-11.6	None
39	ZINC19372622	106.57	-11.6	H-bond
40	ZINC19219257	106.55	-11.6	None
41	ZINC3204340	106.21	-11.6	None
42	ZINC72353749	106.19	-11.7	H-bond
43	ZINC19376358	106.19	-11.8	None
44	ZINC12552475	106.02	-12.1	None
45	ZINC35459261	105.64	-11.7	Salt bridge
46	ZINC13563340	105.48	-11.8	None
47	ZINC21887437	105.37	-11.8	None
48	ZINC257297002	105.31	-12.0	H-bond
49	ZINC257281890	105.18	-11.6	None
50	ZINC19708540	105.16	-11.2	Salt bridge
51	ZINC257219852	105.05	-11.9	None
52	ZINC19708539	104.95	-11.5	Salt bridge
53	ZINC21888194	104.85	-11.9	None
54	ZINC9720915	104.49	-11.1	Salt bridge
55	ZINC72148663	104.39	-11.6	H-bond
56	ZINC3218305	104.07	-11.6	None
57	ZINC257256792	104.04	-12.0	None
58	ZINC17012813	103.96	-11.6	None
ctr	DDD85646	103.59	-11.1	Salt bridge
59	ZINC33252253	103.44	-11.9	
60	ZINC299757953	103.35	-11.3	Salt bridge

61	ZINC19708785	103.31	-11.3	Salt bridge
62	ZINC257322245	103.29	-11.8	
63	ZINC8778846	102.33	-11.6	
64	ZINC72370170	102.19	-11.5	Salt bridge
65	ZINC19710924	102.12	-11.4	Salt bridge
66	ZINC35442574	102.09	-11.6	
67	ZINC17012816	102.04	-11.8	
68	ZINC65074799	101.96	-11.8	
69	ZINC8300432	101.73	-11.6	
70	ZINC257320257	101.54	-11.9	
71	ZINC118915712	101.39	-11.5	Salt bridge
72	ZINC19708784	101.21	-11.6	
73	ZINC8300732	101.12	-11.6	
74	ZINC19017615	101.04	-11.6	
75	ZINC65074799	100.91	-11.7	
76	ZINC8300419	100.87	-12.4	
77	ZINC13959832	100.60	-11.7	
78	ZINC65074790	100.40	-11.6	
79	ZINC230007592	100.36	-11.6	
80	ZINC8300441	100.29	-11.6	
81	ZINC19347017	100.07	-11.9	
82	ZINC257286683	100.05	-11.6	
83	ZINC65074789	100.01	-11.6	
84	ZINC72370169	98.92	-11.5	Salt bridge
85	ZINC2932864	98.78	-11.6	
86	ZINC21881924	98.41	-11.6	
87	ZINC67688793	98.10	-11.4	Salt bridge
88	ZINC72398680	98.01	-11.3	Salt bridge
89	ZINC19219255	97.86	-11.6	
90	ZINC8932149	97.86	-11.8	
91	ZINC20079488	97.75	-11.6	
92	ZINC95354718	97.42	-11.1	Salt bridge
93	ZINC253504901	97.29	-11.7	
94	ZINC19590228	96.79	-11.4	Salt bridge
95	ZINC15064086	96.62	-11.7	
96	ZINC67688794	96.57	-11.2	Salt bridge
97	ZINC257282320	96.45	-11.7	
98	ZINC19374862	96.12	-11.5	Salt bridge
99	ZINC32964503	95.97	-11.6	
100	ZINC19941465	95.86	-11.6	
101	ZINC8300421	95.32	-11.9	
102	ZINC19590227	95.26	-11.6	
103	ZINC8694642	94.93	-11.8	
104	ZINC19566088	94.50	-11.1	Salt bridge
105	ZINC82170861	92.97	-11.7	
106	ZINC91645187	92.93	-11.6	
107	ZINC7738309	88.05	-11.6	
